# Using magnetic resonance imaging to assess visual deficits: a review

**DOI:** 10.1111/opo.12293

**Published:** 2016-04-25

**Authors:** Holly D. H. Brown, Rachel L. Woodall, Rebecca E. Kitching, Heidi A. Baseler, Antony B. Morland

**Affiliations:** ^1^ Department of PsychologyUniversity of YorkYorkUK; ^2^ Hull York Medical SchoolUniversity of YorkYorkUK

**Keywords:** albinism, amblyopia, anophthalmia, functional magnetic resonance imaging, glaucoma, macular degeneration, magnetic resonance imaging, magnetic resonance spectroscopy, ophthalmology, visual deficit

## Abstract

**Purpose:**

Over the last two decades, magnetic resonance imaging (MRI) has been widely used in neuroscience research to assess both structure and function in the brain in health and disease. With regard to vision research, prior to the advent of MRI, researchers relied on animal physiology and human post‐mortem work to assess the impact of eye disease on visual cortex and connecting structures. Using MRI, researchers can non‐invasively examine the effects of eye disease on the whole visual pathway, including the lateral geniculate nucleus, striate and extrastriate cortex. This review aims to summarise research using MRI to investigate structural, chemical and functional effects of eye diseases, including: macular degeneration, retinitis pigmentosa, glaucoma, albinism, and amblyopia.

**Recent Findings:**

Structural MRI has demonstrated significant abnormalities within both grey and white matter densities across both visual and non‐visual areas. Functional MRI studies have also provided extensive evidence of functional changes throughout the whole of the visual pathway following visual loss, particularly in amblyopia. MR spectroscopy techniques have also revealed several abnormalities in metabolite concentrations in both glaucoma and age‐related macular degeneration. GABA‐edited MR spectroscopy on the other hand has identified possible evidence of plasticity within visual cortex.

**Summary:**

Collectively, using MRI to investigate the effects on the visual pathway following disease and dysfunction has revealed a rich pattern of results allowing for better characterisation of disease. In the future MRI will likely play an important role in assessing the impact of eye disease on the visual pathway and how it progresses over time.

## Introduction

Magnetic resonance imaging (MRI) has transformed the field of neuroscience, providing a non‐invasive method to evaluate the structure, function and neurochemistry of the human brain. By correlating brain measures with behavioural outcomes or clinical symptoms, neuroimaging methods such as MRI have allowed researchers to make inferences about the mechanisms underlying differences in clinical presentation.

Approximately 20% of cortex in the human brain is dedicated to visual processing, spanning the occipital lobe and extending into temporal and parietal regions.^1^ MRI can reveal associated changes in the brain, particularly in the visual pathways, to a number of visual disorders, including anophthalmia, glaucoma and age‐related macular degeneration (AMD). In the past, both structural and functional MRI have been used to probe for evidence of reorganisation or degeneration in the human brain as a result of diminished visual input. More recently, magnetic resonance spectroscopy (MRS) has been used to investigate neurochemical changes in visual cortex; changes in metabolite concentrations can then be correlated with structural and functional changes.

In this review, we will focus on the structural, neurochemical and functional MRI modalities and their contribution to our understanding of the way in which the brain responds to visual deficits.

## Visual diseases and disorders

First, we will give a brief overview of several visual diseases and disorders that have been investigated using MR imaging methods. We order the deficits on the basis of an anterior (eye/retina) to posterior (optic nerve/brain) scheme, which we will also use in the review of imaging studies later in the article. Throughout the review we describe studies of individuals with visual field deficits (scotomas), which are typically bilateral thereby removing input to visual cortex. However, we also note where patients have unilateral disease and whether bilateral patients are tested monocularly.

### Anophthalmia and early blindness

Anophthalmia is a rare condition whereby the eyes fail to develop *in utero* and as a result, a child is born without eyes, and there is therefore no means of light stimulation reaching the visual areas of the brain. Anophthalmia is likely to be caused by disturbances of the morphogenetic pathway that controls eye development, either as a result of primary genetic defect or external gestation factors which can influence the smooth process of morphogenesis.^2^ The incidence of anophthalmia is estimated to be 1.8 per 100 000 in the general population.^3^ The study of subjects with anophthalmia provides a novel and scientifically unique group to examine the structure, function and connectivity of the occipital lobe.^4^ We will also review papers on ‘early blindness’. This term captures a variety of causes of visual loss that occur early in development.

### Macular degeneration

Macular degeneration is a family of disorders and causes a progressive loss of central vision by mainly affecting the macula of the retina. Early‐onset manifestations of the disease are usually inherited and are known as juvenile macular dystrophies, or juvenile macular dystrophy (JMD), while late‐onset forms of the disease primarily affect the older population, and are known collectively as age‐related macular degeneration, or AMD. AMD is the leading cause of vision loss in developed countries; in the UK, 12% of all people over the age of 80 are affected by late stage AMD.

Most visual loss occurs in the late stages of AMD due to one of two forms: neovascular, or ‘wet’ AMD or geographic atrophy, or ‘dry’ AMD. Dry AMD is a progressive atrophy of the retinal pigment epithelium (RPE), choriocapillaris and photoreceptors.^5^ Dry AMD is also the most common form of the disease, characterised by a central scotoma caused by a build‐up of drusen in the RPE. Drusen consists of a build‐up of waste products in the eye (such as cholesterol and fatty substances) that would normally be released in the healthy retina. This build‐up damages the photoreceptors, thus leading to central blindness. Dry AMD is a progressive disease for which currently there is no cure.

Neovascular‐AMD (nvAMD) or ‘Wet’ AMD is the more treatable form (most commonly using anti‐vascular endothelial growth factor drugs). In this form, blood, lipids and fluid leak to cause a swelling under the macula. This results in fibrous scarring caused by a breakthrough of new blood vessel growth (choroidal neovascularisation) to the neural retina. This scarring and choroidal neovascularisation damages central vision, progressing to form a central scotoma or distortion of straight lines (metamorphopsia) or both. In the UK, nvAMD accounts for more than half of all cases of registered sight and severe sight impairment.^6^

### Retinitis pigmentosa

Retinitis pigmentosa (RP) is a group of hereditary visual dystrophies, affecting 1 in 4000 individuals, and is the leading inherited cause of blindness.^7^, ^8^ RP is characterised by the progressive loss of both rod and cone photoreceptors and primarily occurs within the peripheral region before developing in the fovea.^9^ Although there is currently no cure for the disease, an intervention study has shown a decline in disease progression following vitamin A and vitamin E supplements.^10^ Recent studies have also examined the potential of retinal implants^11^, ^12^, ^13^ and gene‐therapy^14^, ^15^ to restore visual function.

### Glaucoma

Glaucoma is the second most common cause of blindness worldwide. It is essentially a collection of neurodegenerative diseases caused initially by damage to the optic nerve, affecting both the retina and the central visual pathway.^16^ It can ultimately lead to degenerative changes in the lateral geniculate nucleus (LGN) and visual cortex. However, glaucoma is described as one of the preventable causes of blindness if diagnosed early.

In glaucoma, visual field loss typically starts peripherally. It is often (but not always) associated with elevated intra‐ocular pressure (IOP), causing progressive retinal ganglion cell loss and optic nerve damage resulting in the loss of parafoveal and peripheral vision.^17^, ^18^, ^19^, ^20^ Alarmingly, glaucoma often goes unnoticed and over 50% of ganglion cells may have already died by the time advanced symptoms are present and a diagnosis is made.^21^ Glaucoma has been shown to affect the whole visual pathway, extending from the retina to the optic nerve, LGN and visual cortex, causing deficits involving colour and motion perception, contrast sensitivity and visual acuity.^22^ Primary Open Angle Glaucoma (POAG) is one of the most common forms of glaucoma and will be discussed in this review.

### Albinism

Albinism is a genetic visual disorder, characterised by a lack of pigmentation of the eyes and frequently the skin and hair.^23^ As a result, the eye does not develop normally, causing structural differences, particularly hypoplasia of the fovea.^24^ Moreover, the visual fibres projecting from the retina do not decussate normally, but instead, a larger number (originating from the temporal retina) cross at the optic chiasm. In normally sighted individuals incoming information to each eye is projected to both hemispheres; depth perception and binocular vision both rely on bringing information from the two eyes together.^24^, ^25^ In albinism, both depth perception and binocular vision are impaired. Additionally, the lack of pigment within the iris results in an inability to filter light sources entering the eye, in turn causing photophobia. Nystagmus – the involuntary and sporadic movement of the eyes – occurs alongside albinism, in turn reducing the ability to fixate.^26^ Strabismic amblyopia is also reported to co‐occur with albinism; this is characterised by misaligned eyes and can lead to further visual loss and reduced function if left uncorrected. Given the nystagmus and foveal hypoplasia, people with albinism also have poor visual acuity. It is also of note that achiasma, a condition in which no visual fibres cross the midline, has also been subject to study with imaging methods.^27^

### Amblyopia

Amblyopia is a neurodevelopmental disorder, characterised by monocular visual loss, poor visual acuity, and poor contrast and spatial sensitivity, as shown in psychophysical studies.^28^, ^29^ It is deemed untreatable once in adulthood, but various methods have been used to improve the deficits associated with the disease. Amblyopia is defined in terms of its subtypes: (1) Strabismic amblyopia occurs as a result of misaligned eyes (also referred to as a ‘lazy eye’), and can be remedied somewhat if surgically corrected early in life, but often leads to visual deficits nonetheless; (2) Anisometropic amblyopia (also known as refractive amblyopia) is caused by a large mismatch in focus and refractive power between the eyes, which otherwise appear normal. Less commonly, amblyopia can arise from visual deprivation as a result of cataract.

## Structural MRI

Over the last few decades, advances in MRI technology have allowed for higher resolution images to be captured, with better tissue contrast and spatial resolutions <1 mm^3^. Such advances have now enabled researchers to explore the possible relationship between visual deficits and cortical architecture, particularly within more fine‐grained structures including the lateral geniculate nucleus (LGN) and the white‐matter optic radiations, which may reflect the source or consequence of the deficit.

Within MRI, there are two types of scans often used to measure brain structures – T1‐weighted and T2‐weighted scans – each of which are strongly dependent on magnetic relaxation times. Another MRI technique that has gained popularity recently is diffusion tensor imaging (DTI).

### T1‐weighted imaging

T1‐weighted imaging takes advantage of differences in the time it takes for protons in different tissue types to realign with the main magnetic field (longitudinal magnetisation recovery) following misalignment by a radio frequency pulse. As each tissue within the brain has its own distinct T1‐relaxation time (due to differences in water concentration and movement), this generates a high‐contrast tissue gradient on the MR image, which enables different brain structures to be observed. In T1‐weighted images, cerebrospinal fluid (CSF), with a long T1‐relaxation, usually appears darker compared to the lighter surrounding brain matter, with white matter (short T1‐relaxation) appearing lighter than grey matter.

### T2‐weighted imaging

T2‐weighted imaging on the other hand is based on differences in the time it takes for protons in different tissues to dephase (lose transverse magnetisation). This often produces the opposite tissue contrast to T1‐weighting, whereby CSF (short T2‐decay times) appears lighter compared with the surrounding brain matter (longer T2‐decay). Again, the opposite relationship is also true for grey and white matter contrast, with white matter (longest T2‐decay) appearing darker than grey matter.

### Diffusion tensor imaging

**Figure 1:**
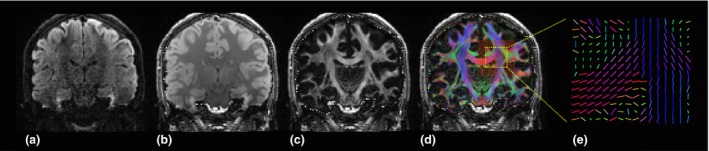
Diffusion measurements reveal white matter tracts. (a) Coronal section through a typical diffusion weighted data volume; dark areas represent areas of relatively high diffusion in a single tested diffusion direction. (b) Diffusion measures are routinely taken in multiple directions. A ‘mean diffusivity’ can be computed by averaging over all diffusion directions as shown in (b); again dark areas represent high ‘mean diffusivity’. (c) This image reveals how directional, or anisotropic, the diffusion is at any given location; areas of high intensity have a high ‘fractional anisotropy’ (FA) and are thus biased for diffusion in a specific direction. (d) The false colour map overlaid on the FA image represents the preferred direction of diffusion at each location; blue colours represent preferred diffusion in the *z*‐plane (superior‐inferior), red in the *x*‐plane (left‐right) and green in the *y* plane (anterior‐posterior). (e) A zoomed representation of the preferred diffusion direction that can be represented by coloured unit line vectors at each location.

Another MRI technique that measures brain structure is DTI (*Figure* 1). DTI works by measuring the 3‐dimensional displacement of water molecules, typically within white matter tracts, in which water diffusion is highly anisotropic due to the movement limitations imposed by neurons, glial cells, axons and other intra‐ and extracellular compartments.^30^ Such measurements can then be used to detect any changes in tissue microstructure between patients and control groups. Quantitative outcome measures include Mean Diffusion (MDi; direction‐independent magnitude of diffusion), Radial Diffusivity (RDi; water diffusion coefficient in a direction perpendicular to the axonal fibres) or, more commonly, Fractional Anisotropy (FA; orientation coherence of water diffusion). Low FA values are thought to indicate the presence of axonal disruption and reduced structural integrity^31^ while high RDi can indicate myelin degeneration and glial cell impairment.^32^

### Voxel‐based morphometry

Initially, MR images (particularly T1‐weighted images) were largely analysed by measuring the absolute dimension of brain regions and white‐matter tracts based on defining anatomical landmarks. However, researchers have begun to examine the local structural composition of individual voxels in order to test for intergroup differences in grey matter, white matter or CSF.^33^, ^34^ This analysis technique, known as voxel‐based morphometry (VBM), can highlight both the extent and location of any structural abnormalities present in patients with visual deficits compared to healthy controls. As a prospective analysis, VBM also benefits from being hypothesis‐ and bias‐free, examining the whole brain without limiting the scope of investigation to specific predefined regions of interest (ROIs).

## Structural MRI in visual deficits

### Anophthalmia and early blindness

Anophthalmic individuals born without fully formed eyes have been studied anatomically with MRI.^4^ At the level of the cortex, only small patches of calcarine cortex exhibited reduced grey matter volume, whereas larger portions of this area of the brain exhibited increases in cortical volume. The largest differences in white matter structure, as assessed volume of white matter, were observed in the optic tract and internal capsule. The optic radiation did not differ from normal in terms of its volume, however its integrity as assessed by FA was significantly lower than normal.

Research on individuals with early blindness have highlighted changes to both white and grey matter structures in the visual system. Anatomical imaging has highlighted atrophy of the optic nerves, chiasm and radiation.^35^ Consistent with the anatomical changes in the optic radiation are the results from DTI studies that also demonstrate atrophy as well as abnormal connectivity within and between the occipital lobes.^36^, ^37^ In addition to the white matter atrophy, cortical atrophy is also evident in early blind patients.^38^ It is clear therefore that congenital and early blindness result in structural changes to the visual pathway. Such changes may underpin the cross‐modal plasticity that is frequently observed in individuals who are blind at an early age.^39^, ^40^

### Macular degeneration and retinitis pigmentosa

Structural abnormalities have also been observed in a variety of hereditary retinal dystrophies, primarily in individuals with Stargardt's disease (a form of JMD) and hereditary RP.

A recent study by Hernowo and colleagues examined whole‐brain and specific regional differences in both white and grey matter between JMD adults and healthy aged‐matched controls. Results showed that JMD patients had bilateral reductions in grey and white matter within visually associated regions, particularly within the occipital poles and LGN, and within the optic radiations compared to controls.^41^ This supports the idea of transneuronal degeneration, whereby the retinal degeneration at the fovea propagates back along the visual pathway and causes atrophy in the retinotopically corresponding areas of cortex. Interestingly, however, a correlation between disease duration and grey matter volume was not found, which is unusual considering the progressive nature of the disease.^41^

The studies of individuals with AMD have revealed very similar findings to those describing JMD. In one study, Boucard *et al*. 42 used VBM to analyse T1‐weighted MR volumes and discovered a significant reduction in grey matter within the posterior region of the occipital lobe in AMD patients compared to aged‐matched controls. In a larger‐scale, multicentre study, Hernowo and colleagues, examined both whole‐brain and regional structure differences in both AMD and JMD patients. They also found bilateral reductions in grey and white matter within both the visual cortex and the optic radiations of the patients.^41^ This showed there are fairly similar structural differences in both JMD and AMD compared to controls, which might be expected given the similarity of visual loss. However, reduced white matter was also found in the frontal cortex in AMD patients but not JMD patients. The authors suggest a possible connection between AMD and Alzheimer's disease, which is currently being investigated.^43^, ^44^, ^45^

Individuals with hereditary RP have also been examined for structural cortical differences. Kitajima *et al*.^46^ found that the calcarine fissure was increased in width in RP patients, notably within anterior and middle regions that normally represent peripheral vision. This corresponds well with the progression of visual deprivation in RP, which starts in the peripheral visual field and progresses inwards towards the fovea. A study by Schoth nearly 10 years later, however, used DTI measurements and found no difference in any white matter tract within the visual pathway, including the optic nerve and corpus callosum, between RP patients and healthy controls.^47^ This may suggest possible differences between white and grey matter abnormalities, although more research is needed to decipher this difference.

### Glaucoma

Glaucoma is one of the most heavily studied visual diseases with respect to MRI structural abnormalities, although the majority of research has been published within the last 5 years. One of the earliest MRI studies in glaucoma was conducted by Kashiwagi *et al.,* 48 who found glaucoma patients (of either primary open‐angle or normal tension subtypes) had a significant smaller optic nerve diameter and optic chiasm height compared to healthy controls. More recent studies have also shown smaller optic nerve and chiasm dimensions in both primary open‐angle glaucoma^49^ and normal tension subtypes.^50^ The LGN has also been shown to be smaller in glaucoma patients using a similar method.^51^ These changes are predicted on the basis of ganglion cell death, but evidence (reviewed below) also indicates significant anatomical changes beyond the LGN.

MR imaging has also been used to assess volumetric changes in both grey and white matter across the cortex. Using VBM, Boucard *et al*.^42^ found that within primary open‐angle glaucoma patients, there were significant grey matter reductions within the anterior and medial occipital lobe. These results are in keeping with the idea of retinotopic‐specific atrophy, whereby only the cortical areas that normally receive input from the damaged part of the retina experience neuronal deprivation. This concept of specific regional loss is supported in the same study in MD patients who, having central‐vision loss, show more atrophy in the posterior occipital lobe.^42^ Other studies have demonstrated clusters of grey matter reduction in glaucoma patients, including bilaterally in calcarine, postcentral and frontal gyri and unilaterally within the precentral gyrus, middle frontal gyrus and within the inferior, middle and superior temporal gyri.^49^, ^52^ Additionally, increases in grey matter were observed bilaterally in the middle temporal, inferior parietal and angular gyri, and unilaterally within the left precuneus and left superior parietal and middle occipital gyri.^49^ Interestingly, however, Li^52^ found such grey matter differences were only significant in severe late‐stage glaucoma and not within the earlier stages where the visual deficit was still non‐existent or limited. This indicates that the extent of grey matter atrophy may be dependent on the level of visual deprivation experienced. However, Williams *et al*.^53^ suggested this relationship might be more complex, with early‐stage glaucoma patients showing increased overall volume within multiple cortical areas (21/93 brain areas studies) compared to healthy controls, while late‐stage glaucoma only showed three larger areas. Nevertheless, 38% of brain regions studied displayed a negative correlation between volume and disease severity, including some regions unrelated to visual processing. The authors suggest that the increases in volume during these early stages may be due to cortical plasticity or be signs of neuronal injury.^53^

Changes in white matter tissue have also been observed along the full length of the visual pathway. In patients with open‐angle glaucoma, white matter volume was reduced bilaterally within the optic nerve, optic tracts, optic chiasm, optic radiations and LGN. Maximum reductions of 78% were found in patients in the optic chiasm and minimum reductions of 16% in the optic radiations compared to aged‐matched controls.^54^

Diffusion tensor imaging methodology has also been used extensively to investigate microstructural differences in visual white matter tracts within glaucoma. Decreases in FA within primary open‐angle glaucoma have been found in the optic nerve, optic radiations, optic chiasm, optic tracts and occipital lobe^55^, ^56^, ^57^, ^58^, ^59^, ^60^, ^61^, ^62^ while increases in RDi and MDi have been found within the optic radiation, optic tracts and optic nerve.^55^, ^56^, ^57^, ^59^, ^62^ The results were replicated in both normal‐tension glaucoma^50^ and within closed‐angle subtypes.^63^ Higher FA and lower MDi levels were found in the optic nerve compared to controls, suggesting that glaucoma patients of different subtypes are likely to display similar structural changes and reduced integrity throughout the extent of the visual pathway. A recent meta‐analysis of DTI studies by Li *et al*.^64^ confirmed that there is an overall significant reduction in FA and increased MDi within the visual white matter pathway in glaucoma patients.

Interestingly, however, Bolacchi *et al*.^61^ found that the location of increased MDi voxels depended on glaucoma severity; increased MDi was present only in the proximal areas of the optic nerve in early‐stage glaucoma patients while both proximal and distal regions were increased in late‐stage glaucoma. A dependence on glaucoma severity was demonstrated also by Garachi^55^ and Dai,^56^ who found that FA values in the visual pathway correlated negatively with glaucoma severity while MDi and RDi values correlated positively. White matter changes, as well as changes found in grey matter by Li^52^ and Williams,^53^ indicate that the severity of glaucoma and corresponding level of visual deprivation can have a substantial effect on brain structure.

### Albinism

The earliest structural MRI study on albinism used T1‐weighted MR images to examine the dimensions of the white matter visual pathways in adults with albinism.^65^ The results indicated that the optic chiasm, optic nerve and corpus callosum all appeared ‘normal’ in size and appearance. However, these initial findings may have resulted from a lack of statistical power as more recent literature has largely found at least some visual pathway abnormalities in patients with albinism. Schmitz *et al.,* 66 using a similar method to Brodsky *et al.,* 65 measured and compared the dimensions of the optic nerve and chiasm within healthy controls and patients and found that individuals with albinism had significantly smaller diameters for both white matter structures. Interestingly, the authors also noted a visual difference in the shape of the optic chiasm, which was described as being more like a ‘x’ in appearance rather than back‐to‐back brackets ‘)(’ seen in controls. This has yet to be verified however, using analysis by blind raters unfamiliar with the participant's group (control or patient). Reduced optic chiasm and optic nerve widths have also been found in other adult cohorts^26^ similar to the adults studied by Schmitz.^66^ Such studies provide evidence using structural MRI for the atypical crossing of optic nerve fibres at the chiasm within albinism, indicating clear pre‐LGN abnormalities.

Post‐LGN structural differences, both in grey and white matter, have also been examined in albinism using MRI. Using T1‐weighted images, Neveu *et al*.^67^ found that the calcarine sulcus in individuals with albinism was significantly smaller in length, depth and surface area compared to healthy controls. Visual cortex abnormalities have also been found by von dem Hagen *et al*.^26^ who, in addition to measuring the optic nerve and chiasm, also used VBM to examined differences in both grey and white matter between individuals with albinism and controls. Their results showed a significant decrease in grey matter volume confined to the occipital pole, while no difference in white matter volumes was observed. Reduced grey matter in individuals with albinism has also been shown in the posterior ventral‐occipital cortex, which also displayed reduced gyrification.^68^ The reduced grey matter in the occipital poles around posterior V1 was attributed to reduced ganglion cell number in the foveal area, due to the underdevelopment of the fovea in albinism.

Interestingly, however, Bridge *et al*. 68 also reported increases in grey matter volume within the superior frontal gyrus and calcarine sulcus, along with an increase in cortical thickness within posterior V1 in individuals with albinism. They theorised that a lack of synaptic pruning within selective cortical areas during development resulted in an increased volume and thickness.

### Amblyopia

More than other visual deficits, there is currently a wealth of research studying structural brain abnormalities within children suffering from amblyopia. This is not entirely surprising, as amblyopia has long been considered a largely cortical phenomenon. Xiao and colleagues compared grey matter volume using VBM in children with either strabismic or refractive anisometropic subtypes. Results showed several clusters of reduced grey matter in the left‐hemisphere middle frontal gyrus, parahippocampal gyrus and inferior temporal gyrus as well as bilaterally in the calcarine cortex within both amblyopic subtypes compared to healthy controls.^69^ Similar results have also been found more recently in amblyopic children, who display clusters of reduced grey matter (left‐hemisphere postcentral gyrus and inferior occipital gyrus and bilateral parahippocampal gyrus) and white matter volumes (left‐hemisphere calcarine, right‐hemisphere precuneus and bilateral inferior frontal areas) compared to control children.^70^ Nevertheless, unlike other studies Li *et al*. (2013) also demonstrate an increase in white matter volume within the right‐hemisphere middle occipital and cuneus areas and within the left‐hemisphere orbitofrontal region. Such increases are thought to represent neuronal plasticity, possibly driven by input from the unaffected eye, resulting in an increase in volume to compensate for the loss of visual input within other areas. Given this result contrasts with other studies this interpretation is quite speculative. Indeed, DTI studies have also revealed microstructural abnormalities within the white matter, with significantly lower FA in the optic radiation of amblyopic children compared to healthy children,^70^, ^71^ particularly in the posterior region,^71^ suggesting reduced structural integrity. Similar indications of reduced structural integrity have also been found more recently in amblyopic adults, who show an increased MDi in the optic radiations, vertical occipital fasciculus, corpus callosum and inferior longitudinal fasciculus.^72^, ^73^

MRI and VBM analysis have also been used to examine structural differences between amblyopic children (with either strabismic or anisometropic subtypes) and their adult counterparts, who were presumably never offered corrective treatment or were treated unsuccessfully as children. Mendola *et al*.^74^ found that, similar to the studies mentioned above, child amblyopes showed reduced grey matter clusters within the visual cortex, specifically in the calcarine and paracalcarine cortices, ventral temporal cortex and both the medial and lateral parietal‐occipital junction. Anisometropic subtypes also displayed further reductions in grey matter compared to strabismic subtypes, who only displayed significant reductions within the right hemisphere. Similarly, adult amblyopes of both subtypes showed reduced grey matter clusters, although less widespread and severe compared to the amblyopic children, although the authors state this could be explained by discrepancies in disease severity arising from recruitment differences between the two groups.

Similarly, Chan *et al*.,^75^ again using VBM on MRI data, discovered decreased grey matter clusters within both visual and non‐visual cortical areas in adults with strabismic amblyopia. This included the calcarine sulcus bilaterally, the intraparietal sulcus, lingual gyrus and cuneus, as well as unilaterally in the superior occipital gyrus and inferior parietal lobule. An increase in grey matter was also observed in the orbitofrontal, frontal, precentral and anterior cingulate regions, again suggesting the possibility of compensatory cortical plasticity effects similar to those found in white matter by Li within children.^70^ Furthermore, this also suggests that higher order visual areas, in addition to the primary areas, may be equally affected by amblyopia. However, a study by Barnes found that while there was significant decrease in grey matter of the LGN, there was no difference observed in either the occipital or temporal lobes between amblyopic adults and controls,^76^ in contrast to results reported by Xiao^69^ and Mendola.^74^ It is possible that the different outcomes in the two studies may be accounted for by methodological differences – while Barnes examined differences in grey matter concentration in functionally‐defined regions, the other studies examined volumetric changes in anatomically‐defined areas.

In another study, Lv *et al*.^77^ used structural MRI to measure differences in regional cortical thickness and found there was no significant difference in either global thickness or regional thickness in primary or secondary visual cortices (V1 and V2) between amblyopes and healthy controls. Nevertheless, patients did show additional hemispheric differences in cortical thickness compared to controls, although the direction of these differences was not stated.

## What role could structural MRI play in the future?

On the whole, research demonstrates that disorders of the eye and visual pathways can have a significant effect on the structural architecture of both grey and white matter in the brain. Both pregeniculate and postgeniculate differences have been revealed within anophthalmia, early blindness, albinism, amblyopia, JMD, AMD and glaucoma, while postgeniculate differences have been observed in RP. Interestingly, such differences are seen not only throughout the visual pathway but also within some areas seemingly unrelated to vision. Furthermore, it is important to note that throughout this literature, there are seeming contradictions in that both increases and decreases in grey matter can be observed within the same condition. Such differences have often been attributed to a wide range of mechanisms including cortical plasticity,^49^, ^53^, ^70^, ^75^ alterations to synaptic pruning^68^ and neuronal damage.^53^ However, these are currently only speculative with no definitive explanation on what such grey matter changes actually represent in terms of structural changes within the brain.

One potential avenue for future MRI research that could shed further light on these mechanisms is to use a longitudinal design to assess the progression of structural abnormality in the posterior visual pathways in relation to deterioration of vision or structure in the anterior aspects of the visual pathway. In the case where retinal damage precedes cortical damage, this would support the idea that structural changes may be directly caused by either loss of functional visual input or through anterograde trans‐synaptic degeneration, where cell death in the retina spreads to connecting neurons to reach different areas of cortex. There is clear evidence for anterograde and retrograde degeneration in the visual system from visual loss due to both eye enucleation and occipital lobe lesioning, respectively.^78^, ^79^, ^80^, ^81^, ^82^, ^83^, ^84^ MRI has the capacity therefore to inform on the timing of such changes resulting from visual loss.

Another potential use for MRI techniques is as a screening method to guide medical management, whereby cortical structural abnormalities could indicate the severity of visual disease or indeed catch it in its earliest stages. Along these lines, a study by El‐Rafei *et al*.^85^ attempted to define a detection mechanism within glaucoma using a classification system constructed from DTI structural data in the optic radiations. The system was able to discriminate between glaucoma patients and controls with 94.1% accuracy and between normal‐tension and open‐angle glaucoma subtypes with 92.8% accuracy. This approach therefore opens up the intriguing possibility of a high performance MRI detection system that could accurately detect the presence of visual disease and possibly replace retinal‐based methodology. However, El‐Rafei did not report the severity of glaucoma patients used; structural differences may only be capable of detecting severe cases, and may only be useful in confirming what ophthalmological techniques had already established, although at a significantly higher monetary cost. Indeed, very few glaucoma studies identified differences between healthy controls and level 0 stage glaucoma (increased intra‐ocular pressure but no visual loss), although some did find structural differences between controls and level 1 (early‐stage glaucoma).^55^ Therefore, while structural MRI may be suitable for assessing severity, it may be unsuitable for early detection of visual diseases, at least for glaucoma.

Structural MRI can also be valuable in guiding the potential direction for treatments and in predicting patient visual outcome after treatment. Although there is currently no cure for many visual diseases, recent treatments have focused on restoring retinal function, through gene therapy,^86^, ^87^ stem cell therapy^88^ and retinal prosthetics.^89^, ^90^ However, there may be a risk to restoring retinal function if brain structure has changed significantly enough to alter the visual pathways and the processing of retinal inputs. Structural MRI may therefore offer a way to determine whether retinal treatments would be effective on a patient‐by‐patient basis, whereby those with significantly altered cortical structure may not benefit from treatment as much as those with greater visual system integrity. Additionally, structural MRI may be beneficial in the development of neuroprotective treatments,^91^ to target the primary areas of neuronal changes and to limit the level of degeneration/atrophy particularly in early stages of visual loss.

In addition to assessing medical treatments, alternative treatments such as behavioural training can be examined through the use of structural MRI. Within AMD, Rosengarth *et al*.^92^ examined the effect of oculomotor training, a common training technique to improve peripheral fixation in those with central vision loss, on brain structure. They found a significant increase in both grey and white matter in the posterior cerebellum compared to those who undertook either no training or vision‐unrelated memory training. While not treating the condition in the same way that medical treatments do, such training might still improve patient independence and increase quality of life.

## Magnetic resonance spectroscopy

Magnetic resonance spectroscopy (MRS) is a technique that allows the chemical composition of the human brain to be measured. As the name suggests the measurement derives a spectrum of resonances that contains peaks at specific frequencies reflecting the different chemicals in the sample. In broad terms, the height of the peaks yields a measure of chemical concentration. Though MRS can be performed using a variety of nuclei, including carbon (^12^C), nitrogen (^14^N), fluorine (^19^F) and sodium (^23^Na), only the nuclei phosphorus (^31^P) and hydrogen (^1^H) exist *in vivo* in concentrations high enough for routine clinical evaluation.^93^ Proton (^1^H) MRS studies have increased in popularity due to the high natural abundance of protons and their elevated sensitivity to magnetic manipulation, better spatial resolution, relative simplicity of technique as well as a shift of interest to areas of metabolism that lack phosphorylated metabolites.^94^ ^1^H‐MRS can be obtained with most MRI systems without additional hardware, provided field homogeneity of the tissue is optimised via a technique known as ‘shimming’.

There are two types of ^1^H‐MRS: single voxel spectroscopy (SVS), which receives the spectrum from a single voxel only or chemical shift imaging (CSI), which measures spectra in projection (1D), on a slice (2D) or a volume (3D). In general, the technique assesses a relatively large (>=8 cm^3^) volume of interest (VOI) in order to maximise signal‐to‐noise ratio. Consequently, a large VOI can present problems as it may include heterogeneous tissue types as well as CSF. The inclusion of CSF in large voxels is particularly problematic in older participants whose sulci and ventricles become enlarged as part of the natural aging process, an issue to bear in mind when investigating metabolic changes in older populations.

### The spectrum

**Figure 2:**
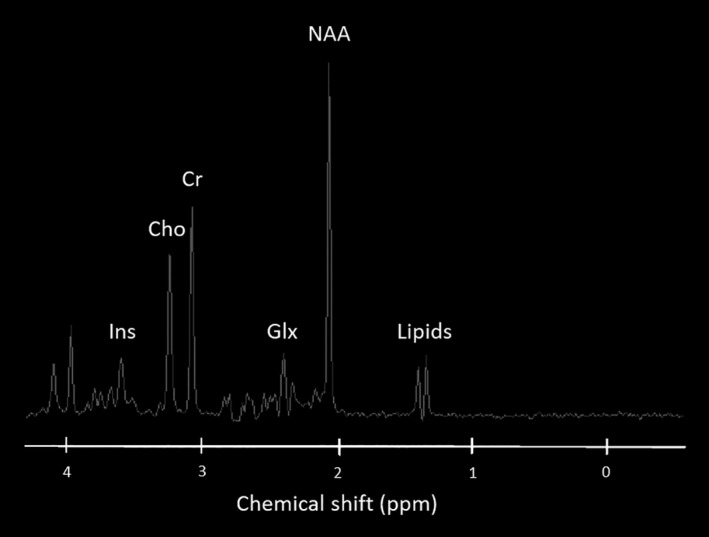
Normal MR spectrum showing the chemical shift in parts per million (ppm) for the metabolites myo‐Inositol (Ins), choline (Cho), creatine (Cr), GABA, Glutamate, Glutamine complex (Glx), N‐acetylaspartate (NAA) and lipids.

^1^H‐MRS data are usually presented as line spectra rather than the two‐dimensional images produced by MRI examinations (*Figure* 2). The peaks on the spectra correspond with various metabolites found within the VOI. The *x*‐axis denotes the chemical shift frequency quantifying the metabolite in parts per million (ppm), and the *y*‐axis plots the relative signal amplitude or concentrations of the various metabolites. Spectra are read from right to left, with the numbers (ppm) on the *x*‐axis increasing in the same direction.

Choline (Cho) represents the total amount of cytosolic choline and is considered a metabolic marker of membrane density and integrity. A decrease in Cho concentration suggests a reduction in cell density, a delay in cell renewal and damage to the signal transport system in neurons.

Creatine (Cr) is known to play an important role in energy metabolism. On the spectrum, the Cr peak actually represents the sum of Cr and phosphocreatine. Tissue death is accompanied by a gradual reduction in Cr, along with other metabolites. In several degenerative diseases, however, Cr concentration levels appear to remain constant throughout the brain. Due to the assumed stability of Cr, clinical evaluations incorporate Cr in order to calculate metabolic ratios such as NAA:Cr and Cho:Cr. However in the research setting, absolute metabolic quantities are measured to increase the sensitivity and specificity of MRS, and regional and individual variability in Cr concentrations have been reported.^94^

**Figure 3:**
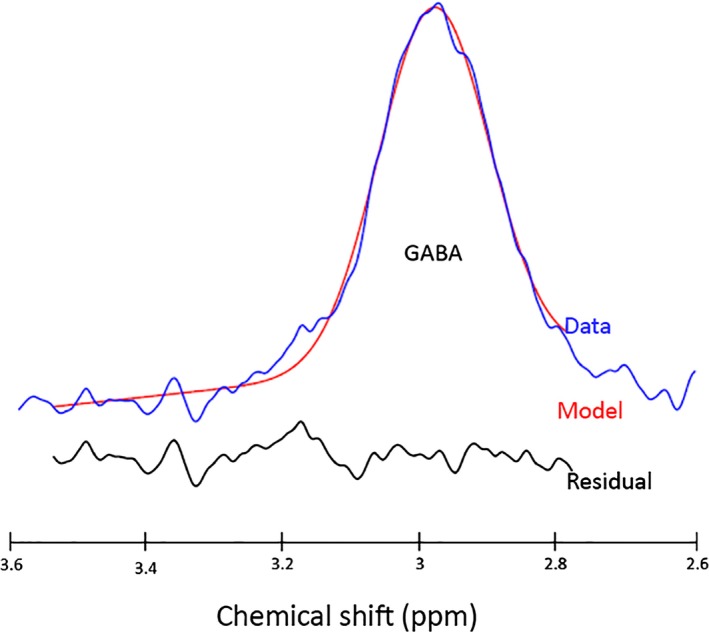
GABA‐edited spectrum produced from a MEGA‐PRESS sequence using the processing tool GANNET. The GABA‐edited data is represented by the blue line with the model of best fit represented in red. The black line below represents the residual between the model and the data.

γ‐aminobutyric acid (GABA), Glutamine (Gln) and Glutamate (Glu) spectral signatures are challenging to isolate. Both Gln and Glu (frequently assessed in MRS in the Glx peak) play a role in detoxification and regulation of neurotransmitters. Glu is the most abundant excitatory neurotransmitter in the brain whereas GABA is the principle inhibitory neurotransmitter. Fortunately, there are methods that can obtain GABA specific measurements from MRS, incorporating J‐difference editing^95^ with the MEGA‐PRESS sequence^96^, ^97^, ^98^ (*Figure* 3).

In terms of brain plasticity, it is believed that GABA is involved in a homeostatic balance between excitatory, inhibitory and modulatory pathways, as shown in animal models of visual disease and visual recovery. This balance mediates developmental and adult plasticity across multiple time scales.^99^ The balance can be shifted towards excitation with up‐regulation of modulatory cholinergic pathways. Similarly, an increase in GABA can shift this balance towards inhibition.^100^

Reduced resting GABAergic inhibition has been shown to trigger ocular dominance plasticity, modulating both the onset and offset of the critical period. Therefore, measuring GABA in response to visual stimulation following deprivation could be a sensitive indicator of plasticity. Short‐term monocular deprivation changes the balance between the two eyes, boosting perception in the deprived eye, reflecting homeostatic plasticity, in which neurons attempt to regulate their own excitability to compensate for sudden changes in input. A study by Lunghi *et al*.^101^ demonstrated that monocular deprivation also induces a concomitant reduction in resting GABA specific to primary visual cortex. This confirms that GABAergic mechanisms for homeostatic plasticity can be demonstrated by combining MRS with psychophysical measures of eye dominance.

*myo‐Inositol* is a simple sugar with a peak at 3.56 ppm. In the brain, *myo*‐inositol is synthesised primarily in glial cells and cannot cross the blood‐brain barrier, and is considered a glial marker. An increase in *myo*‐Inositol concentration is thought to represent glial proliferation or an increase in glial cell size, both of which can occur in inflammation. *Myo*‐Inositol has also been labelled as a breakdown product of myelin.

N‐acetylaspartate (NAA) is found at relatively high concentrations in the human brain parenchyma and is particularly localised within neurons and related to neuronal processes. NAA appears as the highest peak in the normal spectrum, resonating at 2.02 ppm. With good resolution, second and third peaks of NAA can be observed at 2.6 and 2.5 ppm.^94^ NAA is produced in the mitochondria of neurons and transported into the neuronal cytoplasm. A decrease in NAA concentration is routinely considered an indicator of neuronal loss or dysfunction, as such decreases are mostly observed at the moment when a disease is in progress.

## MRS in visual deficits

MRS is an inherently quantitative technique since the signal intensity of a resonance is directly proportionate to a specific metabolite concentration. MRS data can therefore be analysed in conjunction with MRI to correlate anatomical and physiological changes in the brain. For this reason, MRS is a potential tool for studying the metabolic changes in visual cortex *in vivo*, providing direct clinical applications for studying brain plasticity and adaptive changes following sight loss.^102^

To enable effective correlations between MRI and MRS data, it is important to understand any chemical changes that occur in the brain due to the natural aging process, and differentiate them from those occurring as a result of visual disorders. Studies reveal that generally, concentrations of normal metabolites vary slightly with age.^94^ On the whole, by the time a child is 2 years old the spectral pattern will be similar to that of an adult. As the brain matures, the concentration of NAA increases and the concentration of Cho decreases. In the elderly, however, there is a normal decline in NAA.^94^ By taking natural changes due to aging into account, observed changes in metabolic concentration measured in individuals with sight loss can be attributed to the disorder itself.

### Anophthalmia and early blindness

Early blindness is a useful model system for understanding developmental plasticity, as it has been established that when a sense is absent from early life, brain regions usually associated with that particular sense are often recruited by the remaining modalities, a process termed cross‐modal plasticity.^103^, ^104^

Cross‐modal responses and higher levels of functional connectivity^105^ have been found within occipital cortex of early blind individuals. Yet it remains unclear how these cross‐modal changes in functional responsiveness occurring in early blind individuals are mediated. One hypothesis states that changes within occipital cortex in the inhibitory, excitatory and neuromodulatory biochemical pathways drive the changes in functional responses.^106^

Work on animal models has clearly shown that the balance between excitatory and inhibitory neurotransmitters can govern the level of plasticity in the cortex.^107^ Using ^1^H‐MRS to examine the effects of metabolic concentrations in occipital cortex in early blind individuals, a study revealed increased concentrations of *myo*‐Inositol, Cho and Cr and decreased concentrations of GABA.^106^ There were no significant changes in NAA or Glu concentrations. From this, it was concluded that fundamental changes to occipital biochemical pathways occur as a result of cross‐modal responses in early blindness.^106^ However, further work is needed to reconcile these changes with the theoretical framework that can be used to explain how plasticity is underpinned by neurochemical changes.

Anophthalmia is a form of congenital blindness; however, it is not yet clear whether anophthalmia can be thought of as an extreme form of early blindness or whether this disease results in a unique phenomenology.^108^ Studying the effects of visual deficit in this group of individuals is ideal as anophthalmia is not associated with any other neurological impairment. Therefore, investigations in anophthalmia can reveal the effects of complete visual deprivation on healthy brain tissue. Although research has shown large‐scale structural and functional reorganisation of visual cortex as a result of anophthalmia,^109^, ^110^ the neurochemical changes underlying cross‐modal plasticity remain unclear.^109^ Incorporating both MRS and MRI, differences in structure and metabolite concentration were investigated in the pericalcarine cortex in anophthalmic patients.^109^ Results revealed elevated levels of Cho, Cr, Gln, Glu and *myo*‐Inositol relative to sighted controls. Elevated metabolite levels also correlated with significantly greater proportions of grey matter volume. This elevation in metabolic concentration could reflect a shift toward enhanced plasticity or sensitivity that might mediate or unmask cross‐modal responses.^109^

In cases of both early and congenital blindness, research has highlighted evidence of cross‐modal plasticity, whereby brain regions are recruited by other areas to process information. MRS has been integrated with MRI research to show changes that occur chemically as a result of this cross‐modal response. Investigations confirm there is a fundamental change in metabolic concentrations within occipital cortex, including increased levels of Cho, Cr and *myo*‐Inositol, suggesting possible markers of enhanced plasticity.

### Glaucoma and AMD

Retinal damage is in continuous progress in glaucoma and AMD. A number of mechanisms have been invoked to explain the effects of glaucoma, including reactive oxygen species, excitotoxicity, defective axon transport, trophic factor withdrawal and loss of electrical activity.^111^ These pathophysiological actions lead to a series of biochemical compound changes in brain tissue. For example, trans‐synaptic damage caused by excitotoxicity of Glu (as can be assessed by Glx levels) is an important mechanism of glaucomatous central visual pathway injury. Using MRS to investigate metabolic concentrations in the striate area and geniculocalcarine tract, significant decreases in NAA:Cr and Cho:Cr were detected, although there were no reported differences in concentrations of Glx:Cr in a group of glaucoma patients compared with sighted controls.^112^ These results suggest that within the central visual pathway in glaucoma, neurodegeneration is an ongoing process. If progressive visual deprivation affects the metabolism of the adult visual brain, lower concentrations of the metabolite NAA would be expected in occipital cortex, given the association between NAA and neuronal activity. However, one study reported no difference in either Cho, Cr or NAA absolute concentrations in the striate area in a group of glaucoma patients compared to AMD patients, relative to sighted controls.^113^ This indicates that within the visual pathways of the brain, progressive retinal visual field defects do not always induce a measurable decrease in metabolite concentration.

A single MRS VOI can in theory be positioned anywhere in the brain as long as there is limited interference with the metabolic signal. One theory proposed as a factor in glaucoma is the apoptosis theory (programmed cell death). Therefore, one would expect to see an increase in metabolic concentrations of Glu in the vitreous humour and lateral geniculate body (LGB) regions due to the known neurotoxic effects of Glu. MRS was performed using a VOI in these two regions in a group of glaucoma patients. Analysis revealed significantly greater levels of Glx:Cr in both VOIs for the glaucoma patients compared to sighted controls, supporting the apoptosis theory in the aetiopathogenesis of glaucoma.^114^ MRS could therefore move the diagnosis of glaucoma forward enhancing the understanding and diagnosing of glaucoma at the cellular level^114^.

## Summary and future directions for MRS

It is clear that there is a future from MRS in examining the visual system in those with visual deficits. Cross sectional studies have and will shed light on which chemical constituents change in the brain as a result of brief and longstanding visual deprivation. It will also be important to use GABA as a valuable marker of plasticity and take the opportunity to follow such changes over time. The use of higher magnetic field strengths may ultimately improve quantification of metabolite concentrations within the brain and this in turn could help us understand the neurochemistry of visual loss and how it can be harnessed and ameliorated.

## Functional MRI

In addition to its ability to investigate structural and chemical information about the brain, MRI also enables researchers to non‐invasively acquire images of the active brain.^115^ Functional magnetic resonance imaging (fMRI) has quickly become one of the primary techniques used in cognitive neuroscience research, allowing us to investigate how different parts of the brain respond to a given stimulus, and determine how different functions are represented in the brain.

FMRI takes advantage of changes in blood oxygenation levels, and is exploited as a physiological marker of brain activity.^116^ The blood oxygenation level dependent (BOLD) contrast is used to measure the relative amounts of oxygenated and deoxygenated haemoglobin that will vary across the brain depending on the amount of neuronal activity within local regions. This difference in signal in the brain can be measured by acquiring T2*‐weighted images. T2*‐weighting is related to T2‐weighting, but incorporates the additional dephasing effect of the external environment on T2 relaxation times. In sum, an increase in neural activity in a brain region results in a local change in oxygenated haemoglobin, followed by an increase in blood flow to the region, resulting in a local increase in BOLD signal. Visually, this creates an MR image that is brighter in active regions.^117^, ^118^

With regard to vision research, fMRI has been widely used to measure how the brain maps our visual field, and determine how this varies between individuals in health and disease. Before neuroimaging was available, Holmes^119^ outlined key features of how visual space is represented in human visual cortex using brain lesions caused by gunshot wounds. Neuroimaging research has supported his original findings, demonstrating that visual cortex is topographically organised; spatial information is preserved, meaning neighbouring areas in the visual scene are represented by neighbouring neurons in visual cortex.^120^ Moreover, the brain contains multiple maps of visual space, including ‘early’ visual areas V1, V2 and V3. Early visual cortex occupies the medial wall of the occipital cortex; primary visual cortex (V1) occupies the calcarine sulcus, with V2 and V3 bordering V1 both dorsally and ventrally. The central part of our visual field (where visual acuity is highest) is located at the occipital pole and has a much larger representation in visual cortex; this is referred to as cortical magnification. Elsewhere in the visual pathway, the lateral geniculate nucleus (LGN) mediates information coming in from the retina, projecting it to V1. Beyond V1, other visually responsive regions take on higher level processing, for example face and object recognition.^1^

New technologies, such as MR‐compatible eye tracking systems have also allowed researchers to track eye movements and fixation stability in participants. Those with eye disease involving the fovea have difficulty in keeping the eyes fixed on a target in comparison to normally sighted individuals. Eye movements will in turn affect the fMRI data acquired, as stimuli may fall on a different part of the visual cortex in participants with unstable fixation; therefore, when mapping out visual field representations in visual cortex, eye movements could be a confounding factor.^121^ Giving participants a simple fixation task will improve attention and in turn improve fixation stability.

## Functional MRI in visual deficits

Beyond the eye itself, research has shown that after losing sight through retinal damage or disease, regions of cortex dedicated to processing parts of the visual field no longer receiving input are subject to change.^24^, ^25^, ^122^, ^123^, ^124^, ^125^, ^126^ There is a debate in the literature regarding the definition of cortical reorganisation, but also the mechanism by which it occurs, if at all.^127^ Cortical reorganisation can refer to structural changes in the brain, but more frequently, it refers to functional changes whereby patterns of neural activation differ from those observed in healthy individuals. We have proposed that reorganisation should be used only when it is clear that the absence of visual input, combined with the known properties of the normal visual system, cannot explain the pattern of activity observed in patients with visual loss.^127^, ^128^

It has become clear, however, that in addition to ophthalmological measures, such as visual acuity, perimetry, optical coherence tomography and physical examination of the retina, fMRI can aid our understanding as to how the entire visual pathway is affected with eye disease, from retina to cortex.^124^, ^125^, ^129^, ^130^ This will undoubtedly aid further work investigating ways to safely restore vision.

**Figure 4:**
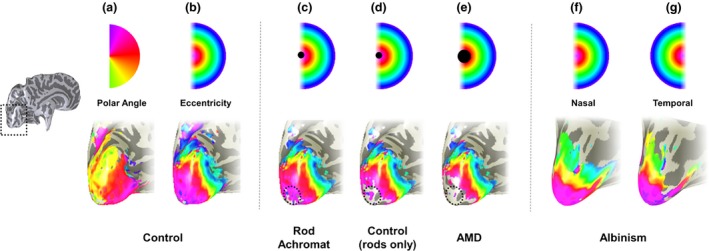
Retinotopic mappings in individuals with eye disease. In each panel, an inflated rendering of the occipital lobe (a magnified version of the area highlighted by the broken line in the hemisphere shown to the left) of the left hemisphere is illustrated. Superimposed on the occipital lobes in false colour are the visual field locations that the brain represents. In each panel the colour coding of the visual field location is given above the visualisations of the brain maps. In (a) and (b), the map of polar angle and eccentricity in a control are given. Polar angle is used to determine boundaries between visual field maps. The eccentricity maps of this control participant are adapted in panels (c–e) to schematise the results observed in individuals with retinal scotomas. In panel (c) a schematic map of eccentricity is shown for a rod achromat alongside, in panel (d), a schematic of a control viewing under scotopic conditions. The broken circle highlights where differences in the map were found; the rod achromat has activity that extends more posteriorly than in the control indicating that the visual cortex has reorganised. In (e), the schematic eccentricity map for an individual with Age‐related Macular Degeneration, in which no evidence of large‐scale reorganisation is shown. In f and g, the eccentricity maps obtained for stimulation of the nasal (f) and temporal (g) retina of the right eye of an individual with albinism are given. Stimulation of the nasal retinal of the right eye produces a map that is very similar to that derived from controls. In contrast to controls however a large representation of the ipsilateral visual field is seen when the temporal retina of the right eye is stimulated. The maps show that a given cortical location responds to equal, but opposite eccentricities.

Typically, fMRI studies include a variety of stimulus types and tasks to tease apart different functions and therefore access information about specific parts of the visual pathway. Retinotopic mapping stimuli are commonly used to define visual maps in the brain.^131^, ^132^, ^133^, ^134^ In the travelling‐wave method, also known as phase‐encoded mapping^132^ participants view two types of flickering checkerboard stimuli: an expanding ring which measures eccentricity (degrees from central fixation), and a rotating wedge which measures polar angle, the dimension that runs orthogonal to that of eccentricity.^131^, ^132^, ^133^ Each stimulus is presented in multiple cycles to cover a portion of the visual field and scans are averaged for wedge and ring stimuli in order to increase BOLD signal‐to‐noise ratio.^135^ As ring stimuli expand and eccentricity increases, activity travels posterior to anterior in the occipital lobes; representations of central vision reside in the occipital pole whereas the periphery is represented in anterior visual cortex (*Figure* 4). Boundaries between each early visual area (separate retinotopic maps) are identified using reversals in polar angle representations^131^, ^132^, ^134^ (*Figure* 4). Retinotopic maps are consistently identified across participants, but can vary in size and anatomical location across individuals.^1^

More recently, population receptive field mapping (pRF) has been used to acquire further information about visual field maps, by modelling the neural response to a variety of stimuli at different positions in the visual field. This allows researchers to estimate the average receptive field size of neurons within a voxel, as well as the mean location represented within the visual field.^136^ Studies reviewed here do not cover pRF methods; however it is of note that this method is increasingly being used in fMRI research.

In addition to visual field mapping techniques, investigators have examined ‘resting state’ fMRI data that allow visual organisation to be probed even in visual regions of the brain that no longer receive or have never received visual input. As the name suggests, resting state scans do not require participants to complete a task or attend to visual stimuli, and so the BOLD response is observed whilst participants are at rest, providing information about cortical regions as well as functional connectivity.^137^ Many studies have used this technique to assess visual field maps in both normally sighted individuals and clinical populations, to discern whether visual representations change as a result of disease. In some instances, retinotopic mapping is not feasible and so other stimuli are used to assess the state of visual cortex; this will be discussed later in relation to eye disease.

### Anophthalmia and early blindness

Cases of anophthalmia and early blindness are valuable particularly when discussing reorganisation.^138^ In order to fully understand how the visual system develops both structurally and functionally, it is important to study individuals who have no visual experience. It has been shown that the organisation of visual cortex, from V1 through to object‐related areas fusiform face area (FFA) and parahippocampal place area (PPA), are retained in individuals with early blindness and anophthalmia.^110^, ^138^ By assessing eccentricity, laterality and elevation – the three main components of retinotopic mapping – the functional connectivity within visual regions could be investigated in the blind. Interestingly, functional connectivity MRI revealed very similar functional divisions in the blind group and the sighted group, thus suggesting that visual input is not fundamental for the development of functional connectivity.^138^

The functional development of visual areas seems to occur through the use of different sensory modalities, such a sound and touch.^139^, ^140^ By examining resting state as well as anatomical connectivity, research has shown that connectivity between the corpus callosum splenium and V1 bilaterally remain intact in individuals with early blindness or anophthalmia.^141^ Furthermore, the occipital lobe in these individuals appears to be incorporated into a functional network for processing language and sounds.^110^ It has been shown that when performing an auditory naming task whereby participants were required to name words described prior, anophthalmics demonstrate greater activity bilateral lateral occipital cortex (LOC) and left V3a when compared to sighted controls. V1 however was activated by any sound, including reversed speech.^110^ This suggests that further into the visual hierarchy, despite processing auditory stimuli, the functional hierarchy remains whereby low level visual areas do not show stimulus preferences, but higher visual areas do. In addition, the calcarine sulcus, posterior temporal, LOC and V5 (a motion processing area) were activated bilaterally by simple tone stimuli.^140^ In addition to increased occipital activity, a reduction in temporal lobe activity was observed in anophthalmics in response to tones.^140^ Overall, individuals with anophthalmia showed more activity within visual cortex to sound.

Studying blindness allows researchers to determine to what extent visual experience aids the formation and development of the visual system. Recent findings suggest that the development of retinotopic organisation is not necessarily driven by retinal waves (action potentials that spontaneously propagate across of the retina before the development of photoreceptors) or visual experience, hence organisation being maintained in anophthalmia.^141^ Overall, evidence from studies including individuals with early blindness and anophthalmia suggests that visual cortex maintains its retinotopic organisation despite having no visual input.

### Macular degeneration

One issue currently under debate within vision research is whether patients with macular degeneration show cortical reorganisation, as a result of losing central vision bilaterally. There is evidence to suggest cortical reorganisation occurs but the mechanism by which this occurs is inconclusive; some researchers have shown that the region of primary visual cortex formally representing the fovea, referred to as the lesion projection zone (LPZ), takes on a new function and processes visual information from the intact peripheral retina.^123^, ^124^, ^126^, ^142^ Some argue that the region of visual deficit must be absolute and include the fovea in order to generate reorganisation, because some patients with foveal sparing do not exhibit reorganisation.^124^ Additionally, the spread of activation in the LPZ is not always large‐scale; in some cases, only part of the occipital pole is activated by stimuli presented to a small, intact part of the retina, suggesting a smaller, more local reorganisation is occurring in these individuals.^142^

After losing central vision, MD patients naturally develop a preferred retinal locus (PRL) in part of the peripheral retina that is still intact. Typically, a PRL will develop in a region abutting the scotoma, but the location depends on the size and extent of the damaged retina.^143^, ^144^ It is thought that the establishment of a PRL may trigger reorganisation, as it essentially takes on the function of the formerly intact fovea.^143^ However, while some participants exhibit LPZ activity to a greater extent for stimuli presented to the PRL compared to a peripheral location of equal eccentricity (‘non‐PRL’),^143^ others have argued there is no difference between peripheral locations.^126^ Any subtle differences between the PRL and non‐PRL are thought be driven by attention.^126^

In contrast, some researchers, who used retinotopic mapping methods, report a lack of reorganisation in patients with MD and JMD as no discernible activity was detected in the LPZ in all cases.^122^, ^125^, ^145^ This contrasts with the remapping of retinal input revealed in retintopic mapping experiments on rod achromats, who have a small central scotoma at birth^146^ (for comparison see the schematics in *Figure* 4). The absence of remapping in the patients with AMD and JMD was also confirmed by modelling the expected cortical responses on the basis of the retinal scotoma.^125^ A lack of reorganisation may be positive news with respect to treatment restoring retinal function; if cortex has not taken on a new role it is theoretically ready to resume processing incoming information (although see earlier sections on the atrophy of the LPZ).

It is also of note that the ‘resting state’ activity in the LPZ has been probed to understand how visual organisation might change in this zone which no longer receives input.^147^ This adds weight to the idea that the organisation of the visual cortex is largely unchanged by a partial removal of input, consistent with the organisation found in individuals, who have no visual input from birth.^4^, ^110^

Other studies have investigated apparent activity in the LPZ in terms of feedback from extrastriate visual areas.^129^, ^130^, ^148^ Using multiple stimulus types (including faces, checkerboards, drifting contrast patterns) in two different conditions; a one‐back task and passive viewing, stimulus‐synchronised responses were apparent within the LPZ in 75% of participants when performing a task. Under passive viewing conditions, stimuli elicited zero or negative responses in the LPZ.^129^ Task‐dependent activity in the LPZ was also shown in patients with RP.^130^ The authors suggest that LPZ activity might represent the unmasking of a normal feedback mechanism from extrastriate regions, due to task demands. However, age‐matched, normally‐sighted control participants did not exhibit responses in the unstimulated region in either passive or task conditions. Li *et al.,* 148 refer to this feedback mechanism as a form of functional reorganisation.

The majority of research described above includes both forms of MD, both age‐related and juvenile. Research has shown that while qualitatively JMD and AMD are quite similar regarding neural responses and extent of reorganisation, the size of the silent part of the LPZ is noticeably smaller in JMD, even when scotoma size is accounted for.^148^ It is assumed that in MD, the visual system developed typically prior to disease onset. The slight difference between may represent the time course of plasticity and emphasise that earlier onset allows for increased plasticity.

Visual hallucinations are a common symptom of Charles Bonnet Syndrome, found to co‐occur with MD in some cases. It is worth noting that fMRI has also been used to investigate the possible neural mechanisms underlying this symptom,^149^, ^150^, ^151^ however this beyond the scope of this review, and so it will not be discussed further.

### Glaucoma

Structural changes have been observed as discussed previously, but comparatively fewer studies have examined the functional changes associated with glaucoma.^152^, ^153^ As with all eye diseases, it is important to understand how the brain is affected, in accordance with behavioural and perceptual deficits. The following studies explore both bilateral^22^, ^153^, ^154^ and unilateral^22^, ^155^, ^156^ glaucoma. In bilateral cases, the fellow eye is often less affected than what authors refer to as the glaucomatous eye. Animal work has previously shown a multitude of changes associated with glaucoma, including neurochemical modulations in the ocular dominance columns for both the affected and the unaffected eye, a reduction in GABA levels which in turn causes reduced inhibition and thus a greater response to the unaffected eye in cortex.^155^ The question has been raised as to whether fMRI can provide evidence of cortical reorganisation in patients with glaucoma, and determine to what extent of functional plasticity occurs. FMRI studies have used various checkerboard stimuli to map visual cortex in glaucoma^132^, ^134^, ^153^, ^156^; standard retinotopic mapping is not always ideal because the representation of scotoma in early visual cortex can be difficult to map accurately and so a template fitting approach has been adopted using static checkerboard stimuli.^153^, ^157^ Other stimuli used (static or with contrast reversal) include full field checkerboard, radial checkerboards occupying a hemifield^22^, isopter (arc), ‘bow tie’ and ‘hourglass’ shapes (comprising checkerboards) to map the scotoma, and vertical and horizontal meridians respectively.^22^, ^153^, ^156^ Using these methods, studies have reported a reduction in the BOLD response within V1.^153^, ^156^ Monocular testing revealed similar maps for the glaucomatous and the unaffected eye, with matching numbers of active voxels, however responses were weaker in contrast to the unaffected eye.^22^, ^156^ The biggest reduction of the BOLD response occurred whilst stimulating the glaucomatous eye with high contrast stimuli; this may suggest a particular deficit in the parvocellular pathway, however direct investigation is required to address this question.^156^ The results highlight the sensitivity of fMRI in detecting the scotoma and corresponding cortical responses. Despite the change in the magnitude of the response, which can be explained by reduced visual acuity, the fact the organisation and general size of the maps do not change significantly between the eyes suggests a stable functional organisation in visual cortex in patients with unilateral glaucoma.^156^

Functional magnetic resonance imaging is an important tool for glaucoma research as the disease in known to extend beyond the eye; fMRI responses have been shown to correspond to the extent of visual loss and correlate with standard ophthalmological measures such as perimetry.^22^ Understanding these global changes is important particularly because there is evidence to suggest that different types of glaucoma impact on cortex differently. Previous studies focussed on patients with primary open angle glaucoma (POAG), but a study on normotensive glaucoma found no discernible change in functional activity in visual cortex.^154^ Overall, this will inevitably improve our understanding of the disease and the mechanism underlying the functional deficits, alongside any structural abnormalities.

### Albinism

Research in both animals and humans has shown dysfunction in the visual pathway of individuals with albinism expressed as an abnormal crossing of retinal fibres.^23^, ^24^, ^25^, ^26^, ^158^, ^159^, ^160^, ^161^, ^162^, ^163^

Monocular stimulation in participants with albinism reveals that the representation of the nasal retina matches that of normally sighted control participants, yielding responses in the contralateral hemisphere.^23^, ^24^, ^25^, ^163^ However, cortical responses to temporal retinal locations are abnormal. The same regions in visual cortex contralateral to the stimulated eye respond to the temporal retina, as well as nasal retina^24^, ^25^ (*Figure* 4). Additionally, as eccentricity increases, ipsilateral regions take over and representations of temporal retina begin to resemble those of healthy sighted controls again.^24^ This suggests the representation of the temporal and nasal retinal is mirrored and a single voxel is processing two parts of the visual field of comparable eccentricity. As referred to above, the cortical representation reverts to normal at a specific eccentricity – the line of decussation. It has been shown that the shift in the line of decussation is linked to the pigmentation level in individuals with albinism.^164^

The standard method of diagnosing albinism involves measuring visually evoked potentials.^26^, ^161^, ^165^ Using full field stimulation, deficits regarding hemispheric lateralisation were observed, whereby the hemisphere contralateral to the eye being stimulated responded more strongly.^26^ The spatial resolution of fMRI appears to allow albinism to be detected with greater sensitivity and specificity.^26^ In the case of albinism, therefore, diagnosis may be improved with the addition of fMRI. Researchers have shown fMRI responses to full field stimulation highlight differences in lateralisation of responses between albinism and controls (healthy sighted and individuals with nystagmus). However, responses to hemifield stimulation are similar between groups with albinism and those with nystagmus, but when restricted to the most posterior slices of the occipital lobe, a group difference emerged.^26^

It is also interesting to note that a rare condition, achiasma, meaning that no descussation of the visual fibre occurs, results in very similar cortical organisation patterns to those found in albinism.^166^, ^167^

### Amblyopia

Amblyopia has been studied extensively as its neural basis is still a source of debate. The advantage of studying amblyopia in particular is that it allows for the examination of reorganisation within the visual pathway which is dependent on experience.^77^ Multiple neuroimaging studies have demonstrated that V1, extrastriate cortex and the LGN are affected in amblyopia.^168^

#### Striate cortex (V1)

Researchers initially debated whether deficits in V1 were the source of behavioural deficits observed.^168^, ^169^, ^170^ V1 comprises ocular dominance columns, made up of neurons that respond to input from one eye, or the other. Previous work in animals demonstrated that amblyopia results in a shift in ocular dominance, suggesting that the deficit is underpinned by changes to the brain.^171^, ^172^ However, early human post‐mortem studies showed no evidence of anatomical changes in ocular dominance columns.^173^, ^174^ More recent work in humans utilising fMRI have informed on this debate. Stimuli consisting of sinusoidal gratings, drifting at high contrast are ideal for exciting visual cortex.^175^, ^176^ An early fMRI study in humans examined changes in ocular dominance columns, and found more voxels representing the unaffected eye.^176^ This study also examined differences between early and later onset amblyopia, however this is a controversial finding and further investigation is still required.

Further work has been conducted to map out visual cortex in both strabismic and anisometropic amblyopes, using standard retinotopic mapping procedures described previously^121^, ^177^ to map visual representations in each eye independently.^121^ A reduction in fMRI signal was observed in representations of the amblyopic eye, compared to the unaffected eye; however this result was not consistent across participants. While on average amblyopes demonstrated a larger difference in ocular dominance compared to controls, they also showed a smaller region of cortex representing either eye.

To determine if subtypes of amblyopia have differential effects on visual cortex, studies have examined responses to various checkerboard stimuli, manipulating the size and number of reversals to tease apart the differences.^170^, ^178^ Results indicate that in strabismic amblyopes, there is a reduction in activity within the calcarine sulcus in response to stimuli of lower spatial frequency. In contrast, anisometropic amblyopes show a reduction in calcarine activity in response to high spatial frequencies.^170^ Most studies do not report a significant difference between subtypes and therefore combine participants for analysis.^29^, ^77^

#### Extrastriate

While many studies have focussed on the effects of amblyopia on early visual areas by stimulating the eye with simple achromatic pattern stimuli,^169^, ^175^, ^176^ it is important to assess deficits extending into extrastriate visual areas.^179^ Behavioural measures have not been shown to correlate with activity in striate cortex in amblyopia, suggesting perhaps deficits correlate with activity in higher visual areas.^121^ Face and scene processing have been investigated in both strabismic and anisometropic amblyopes but results show no significant differences between types of amblyopia.^180^ Under monocular viewing conditions, a decrease in activity in face‐related cortical areas was evident in response to faces when viewing with the affected eye, but no such deficit was shown in building‐related cortical areas in response to building stimuli. Poor visual acuity in the amblyopic eye and the fact that viewing faces requires processing of finer details may explain why patients appear less proficient at face processing as well as reduced neural responses in face processing regions.^181^, ^182^ Behavioural testing of the amblyopic eye also demonstrated a larger deficit in identity recognition and to a greater extent, facial expressions, performing better with naming and categorising building stimuli.^180^ However, given that neural responses to building stimuli also appear normal, suggests a specific deficit within the ventral stream.^177^, ^179^ The higher level deficit is also dependent on the size and position of the stimulus; when smaller stimuli were presented to the amblyopic eye, the deficit remained, however for larger stimuli, responses were more similar to those observed with the unaffected eye^179^ This was apparent in V1 through to V4 and also in the posterior fusiform gyrus.

In addition to ventral stream abnormalities, research has revealed a deficit in V5/MT in ambylopes, suggesting the dorsal stream is also compromised in amblyopes.^183^, ^184^, ^185^ Global motion deficits have primarily been tested psychophysically, however fMRI studies have also shown reduced activation in motion areas. Anisometropic amblyopes show reduced activity in V5 as well as V3a, which authors suggest is not attributed to poor visual acuity or blurring, as control participants did not show a deficit in motion processing areas following monocular blurring or by decreasing the luminance.^185^ Strabismic amblyopes also show a similar deficit but to a greater extent that anisometropic, particularly for the high‐level random dot kinematograms.^183^ The cause of this reduction in motion areas is not clear however structural imaging has shown a reduction in grey matter in amblyopes.^74^ It is also of note that the areas recruited when processing motion are no different between the amblyopic and non‐amblyopic eyes, it is the magnitude of the response within motion areas that differs.^184^

The studies above do not rule out the possibility that the observed deficits in higher visual areas are the result of deficits in earlier visual areas. In addition to examining responses in different visual areas following eye disease, it is important to establish if the areas themselves are affected or whether it is the connections between areas where the deficit occurs.^186^ The effects of amblyopia have been investigated in terms of both dorsal and ventral processing, focusing on the connectivity between striate and extrastriate visual areas. ROIs included the LGN and V1 to V4 (defined retinotopically).^186^ In addition to establishing the degree of connectivity, researchers can assess directional effects along the thalamo‐cortical pathway. *Table* 1 summarises the directional effects within visual cortex.^186^ Any significant reductions in activity have been indicated with a downward arrow. Overall there is a significant reduction in feedforward and feedback connectivity for the amblyopic eye. A significant reduction in feedforward connectivity between the LGN and V1 was observed, particularly in the ipsilateral hemisphere, whereas feedback connectivity was impaired in both hemispheres.^186^

**Table 1:** Reduction in connectivity in dorsal and central processing streams in amblyopia, as shown by Li *et al*.^186^ Downward arrows indicate significant reduction in connectivity

Connection	Ipsilateral hemisphere		Contralateral hemisphere	
LGN → V1	↓			
LGN ← V1	↓		↓	

Connection	Ventral stream		Dorsal stream	
	Ipsilateral hemisphere	Contralateral hemisphere	Ipsilateral hemisphere	Contralateral hemisphere
V1 → V2	↓	↓	↓	↓
V2 → V4	↓			
V1 → V4	↓	↓		
V1 ← V2	↓			
V1 ← V4	↓	↓		
V2 ← V4	↓	↓		
V1 → V3a				↓
V2 → V3a			↓	
V1 ← V3a				↓
V2 ← V3a			↓	↓

When V1 was excluded from analysis, connectivity was still significantly reduced in both hemispheres overall, suggesting that any V1 changes may not fully account for the deficit. Overall, the study demonstrated that when the amblyopic eye is stimulated, there was a significant impact on both feedforward and feedback connectivity within the visual pathway, in both the dorsal and ventral streams. The change in connectivity correlated with behavioural deficits, but did not correlate with the fMRI signal in visual regions.

#### Lateral geniculate nucleus

The role of the LGN in amblyopia has been explored to determine whether deficits are apparent earlier in the visual pathway.^29^, ^152^ The LGN can be localised using both structural and functional imaging; showing participants checkerboard stimuli of high contrast allows for functional localisation. A reduction in LGN activity was observed in all participants for both hemispheres when the amblyopic eye was stimulated, relative to the unaffected eye. However there were no hemispheric differences when viewing with the amblyopic eye or the unaffected eye.^29^ Similar responses across hemispheres suggest there is no difference with regard to the input to the LGN.^29^ This work is important as it stresses that deficits are not restricted to cortex. Given that the LGN plays an important role in the development of vision, it is possible that its role in amblyopia is simply that it does not moderate the visual system in the typical manner.^187^ It is yet to be determined whether the deficits observed in the LGN are a result of local influences within the LGN itself, or whether it is a result of abnormal connectivity with cortex.^29^, ^186^

Amblyopia is deemed untreatable in adulthood. However, different therapies are being investigated to alleviate some of the deficits. Levodopa has been piloted as a potential treatment for amblyopia.^188^, ^189^ Another approach is to apply transcranial direct current stimulation (TDCS) to visual cortex^28^. We acknowledge that there is ambiguity within the literature regarding the effectiveness of therapies; however, it is beyond the scope of this review and therefore will not be discussed. In the case of any treatment, however, it would be valuable to use fMRI (and potentially other MR methods) to provide visual cortical biomarkers of treatment outcome.

## Conclusions

This review has examined how magnetic resonance imaging (MRI), including structural, spectroscopy and functional scans, has been utilised to investigate a selection of visual disorders. Structural MRI has revealed abnormalities in grey and white matter density across both visual and non‐visual areas in conditions including amblyopia, albinism, glaucoma and macular degeneration. Structural MRI has been used in numerous studies particularly in glaucoma, where a reduction in visual pathways has been detected. Studies have shown similar reductions in grey and white matter in the posterior occipital lobe and optic radiations in both age‐related macular degeneration (AMD) and juvenile macular degeneration (JMD). Patients with AMD also displayed reduced white matter in the frontal cortex, supporting the association between AMD and Alzheimer's disease. Although there have been relatively few studies on RP, studies have shown deterioration within anterior and middle occipital lobe but have not revealed any abnormalities earlier within the visual pathway. The overriding conclusion therefore is that the visual cortex undergoes changes following loss of functional inputs. It remains to be seen whether the changes are permanent and irreversible, which may impact on treatments for visual restoration. It will also be important to assess what the nature of the atrophy is – does it involve cell death and or significant rewiring. Current methods are unlikely to inform on these issues as yet, but future research in this area is important.

MR Spectroscopy is at an earlier stage than MR imaging approaches in terms of assessing visual deficits. However, the research in this area has yielded some important preliminary observations. Importantly, the recent advances in measuring GABA – a key neurotransmitter that plays an important role in vision and cortical plasticity – will provide new and important ways to investigate visual deficits.

Functional MRI provides evidence of extensive functional changes throughout the visual pathway as a result of vision loss. It is still not entirely clear if functional reorganisation occurs with AMD and JMD, or whether activity within the lesion projection zone, representing the scotoma, is a result of normal feedback mechanisms, mediated by task demands.^123^, ^125^, ^129^ Relatively fewer studies examine the functional changes in glaucoma, but the evidence that there are silent areas in visual cortex representing the scotoma, provide little indication of cortical reorganisation. In albinism, abnormal retinogeniculate projections do not appear to reorganize, instead reorganisation is likely at a synaptic level to allow for the normal and abnormally projecting information to co‐exist in cortex.^24^ The visual pathway is affected significantly in amblyopia, irrespective of subtypes. As well as specific deficits within LGN, V1 and extrastriate areas, investigations of amblyopes reveal evidence of reduced functional connectivity, including both feedforward and feedback mechanisms.^29^, ^186^

Besides informing on the way in which different diseases affect the visual pathway, from retina through to visual cortex, MRI could be used to aid diagnosis, and determine the potential course and efficacy of treatments. Although there is currently no cure for the majority of visual diseases, recent treatments have focused on restoring visual function via a variety of approaches (stem cell therapy, gene therapy, retinal prostheses, etc.). However, the success of retinal restoration depends on the extent to which the rest of the visual pathway function and structure have remained stable from the point sight was lost, without any significant degeneration or reorganisation. MRS could also be incorporated to predict successful restoration of vision by measuring levels of inhibitory neurotransmitters before treatment. If there is evidence of reorganisation, functional or structural, this will impact how the new information entering the retina is processed in all elements of the visual system.

By examining functional activity, researchers can determine whether changes are occurring and over what time course. MRI can therefore be used to determine whether retinal treatments would be effective on a patient‐by‐patient basis, perhaps excluding those with significantly altered cortical structure that may not benefit from the treatment. Additionally, structural MRI may also be used to guide the development of neuroprotective treatments,^91^ which could be used to target the primary areas of neuronal changes, potentially limiting the level of degeneration particularly in early‐stages of visual loss. Finally, MRS shows great promise as an assay for detecting biomarkers indicating plastic or degenerative changes associated with visual disease.

## Disclosure

The authors report no conflicts of interest and have no proprietary interest in any of the materials mentioned in this article.
